# The Evolution of Ovarian Carcinoma Subclassification

**DOI:** 10.3390/cancers14020416

**Published:** 2022-01-14

**Authors:** Martin Köbel, Eun Young Kang

**Affiliations:** Department of Pathology and Laboratory Medicine, University of Calgary, Calgary, AB T2N 2T9, Canada; eykang@ucalgary.ca

**Keywords:** ovarian cancer, subclassification, histotype, molecular subtype, immunohistochemistry

## Abstract

**Simple Summary:**

Historically, cancers presenting with their main tumor mass in the ovary have been classified as ovarian carcinomas (a concise term for epithelial ovarian cancer) and treated with a one-size-fits-all approach. Over the last two decades, a growing molecular understanding established that ovarian carcinomas consist of several distinct histologic types, which practically represent different diseases. Further research is now delineating several molecular subtypes within each histotype. This histotype/molecular subtype subclassification provides a framework of grouping tumors based on molecular similarities for research, clinical trial inclusion and future patient management.

**Abstract:**

The phenotypically informed histotype classification remains the mainstay of ovarian carcinoma subclassification. Histotypes of ovarian epithelial neoplasms have evolved with each edition of the WHO Classification of Female Genital Tumours. The current fifth edition (2020) lists five principal histotypes: high-grade serous carcinoma (HGSC), low-grade serous carcinoma (LGSC), mucinous carcinoma (MC), endometrioid carcinoma (EC) and clear cell carcinoma (CCC). Since histotypes arise from different cells of origin, cell lineage-specific diagnostic immunohistochemical markers and histotype-specific oncogenic alterations can confirm the morphological diagnosis. A four-marker immunohistochemical panel (WT1/p53/napsin A/PR) can distinguish the five principal histotypes with high accuracy, and additional immunohistochemical markers can be used depending on the diagnostic considerations. Histotypes are further stratified into molecular subtypes and assessed with predictive biomarker tests. HGSCs have recently been subclassified based on mechanisms of chromosomal instability, mRNA expression profiles or individual candidate biomarkers. ECs are composed of the same molecular subtypes (*POLE*-mutated/mismatch repair-deficient/no specific molecular profile/p53-abnormal) with the same prognostic stratification as their endometrial counterparts. Although methylation analyses and gene expression and sequencing showed at least two clusters, the molecular subtypes of CCCs remain largely elusive to date. Mutational and immunohistochemical data on LGSC have suggested five molecular subtypes with prognostic differences. While our understanding of the molecular composition of ovarian carcinomas has significantly advanced and continues to evolve, the need for treatment options suitable for these alterations is becoming more obvious. Further preclinical studies using histotype-defined and molecular subtype-characterized model systems are needed to expand the therapeutic spectrum for women diagnosed with ovarian carcinomas.

## 1. Introduction

**Figure 1:**
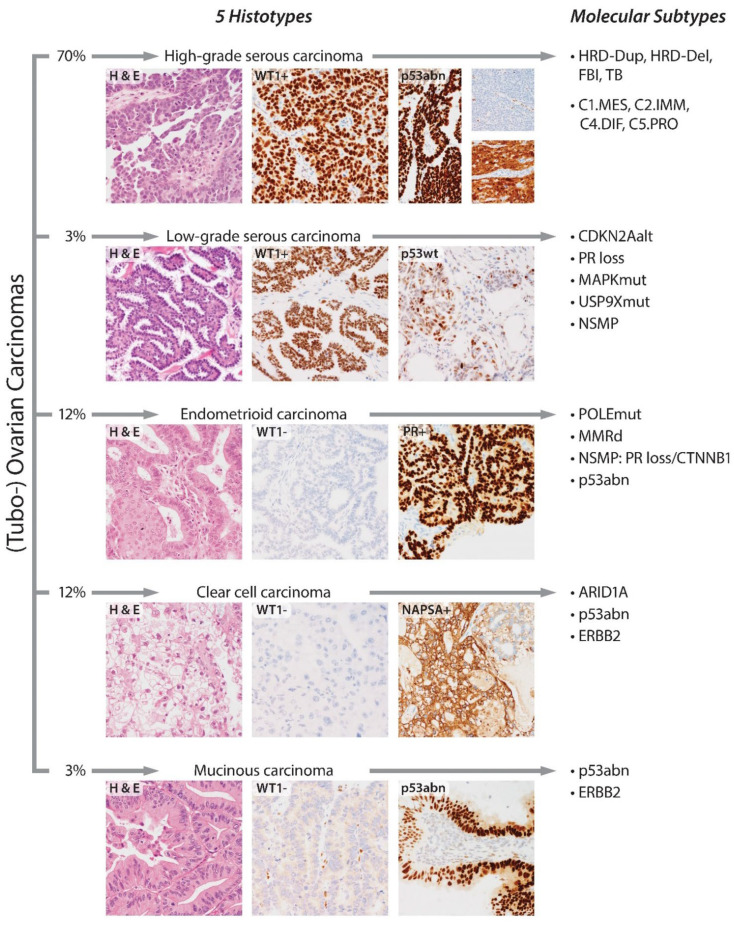
Stratification of (tubo-)ovarian high-grade serous, low-grade serous, endometrioid, clear cell and mucinous carcinoma histotypes into molecular subtypes. NAPSA = napsin A; HRD = homologous repair deficiency; Dup = *BRCA1*-associated tandem duplications; Del = *BRCA2*-associated interstitial deletions; FBI = fold-back inversions; TD = tandem duplications; *CDKN2A*alt = *CDKN2A* alterations; MAPKmut = MAPK pathway mutations; *USP9X*mut = *USP9X* mutations; NSMP = no specific molecular profile; *POLE*mut = *POLE* mutated; MMRd = mismatch repair deficient; p53abn = p53 abnormal; p53wt = p53 normal/wild type.

The subclassification of ovarian carcinomas is now based on a hierarchical approach; the first step is to subclassify based on traditional histopathological phenotypes into histotypes. Histotypes are considered different diseases based on the cell of origin, molecular alterations, clinical behavior and management [1,2]. Precise histotyping is now supported by ancillary diagnostic immunohistochemical (IHC) markers [3]. Although refined by molecular advancements, the phenotype-based histotype classification has been relatively stable over several decades. In the second step, histotypes are then further stratified into molecular subtypes (Figure 1). Molecular subtypes and predictive biomarker tests are currently evolving.

The term “ovarian carcinoma” (concise for epithelial ovarian cancer) has become somewhat problematic because it may not correctly reflect the site of origin and may serve as an umbrella term that includes other primary sites, such as the fallopian tube and peritoneum. Therefore, a histotype-specific approach is more appropriate. For high-grade serous carcinomas (HGSCs), there is overwhelming evidence that the majority arise from a precursor within the fallopian tube: serous tubal intraepithelial carcinoma (STIC) [4,5]. Pathology-reporting guidelines have recently changed to reflect this [6]. HGSCs are now assigned as tubal origin when the fallopian tube is involved either by STIC or mucosal carcinoma, or if the fallopian tube is overgrown by HGSC. Hence, the majority of HGSCs are now considered extraovarian in origin. A dramatic shift in the IDC-O site codes from C56.1 (ovary) to C57.0 (fallopian tube) can be expected in the upcoming years. Given the biological and clinical similarities, the 2020 fifth edition of the WHO Classification of Female Genital Tumours now uses the combined terminology of *tubo-ovarian* high-grade serous carcinoma [7]. With changes in site assignment, primary peritoneal high-grade serous carcinoma is now exceedingly rare. Although the other histotypes are generally assigned to an ovarian primary, endometrioid (EC) and clear cell carcinoma (CCC) arise from endometriosis, which is ectopic endometrium, meaning that the tissue of origin is not the ovary [8,9]. Low-grade serous carcinomas (LGSCs) are of fallopian tube-type cell lineage, are often meta- or synchronously associated with serous borderline tumors and show frank invasion in the ovary (ovarian primary). However, some can show frank invasion in the peritoneum (peritoneal primary) or even in lymph nodes (lymph node primary) [10]. Notably, a reproducible assessment of invasion at a peritoneal site is challenging, and the frequency of peritoneal LGSC differs between centers, which might, in part, explain the survival differences for patients diagnosed with peritoneal versus ovarian LGSCs, with the former having a longer survival [11,12]. If metastatic adenocarcinomas (mostly from the lower or upper gastrointestinal tract) are excluded, ovarian mucinous carcinomas (MCs) arise from the ovary. However, despite their obvious progression from benign/borderline to malignant, a convincing normal cell of origin in the ovary remains elusive. Rare cases are associated with Brenner tumors or are of germ cell origin (associated with teratomas) [13,14].

## 2. Evolution of Histotypes

The current 2020 fifth edition of the WHO Classification of Female Genital Tumours lists six main histotypes (also referred to as histological types and cell types, formerly subtypes) and four other histotypes in the category of ovarian epithelial neoplasms [7,15]. Seven were already listed in the first edition published in 1973, demonstrating that the phenotype-based histotype classification is relatively stable (Table 1) [16].

However, a major change was introduced in 2014 with the fourth edition [17]. Based on the discovery by Kurman, Shih and colleagues that serous carcinomas follow a dualistic pathway of development, with low-grade tumors harboring mutations in the MAPK pathway (*KRAS*, *BRAF*, *NRAS* and others) versus high-grade serous carcinomas now ubiquitously characterized by *TP53* mutations, serous carcinomas were divided into LGSCs and HGSCs as separate histotypes and not only a continuum of grade [18,19]. The clinical management of these two histotypes is now different, highlighting the importance of accurate diagnosis [20].

When comparing the pre-2014 WHO classification with the post-2014 standardized pathology review, the main changes over time were the reclassification of a significant subset of endometrioid, undifferentiated and unclassified carcinomas to high-grade serous carcinomas [21,22,23]. This was based on the understanding that these tumors are molecularly similar to HGSCs and show expression of WT1 as a diagnostic marker, and the recognition that high-grade serous carcinomas can show morphological features of endometrioid or undifferentiated carcinomas (so-called “SET features”—solid, pseudoendometrioid/glandular and transitional cell carcinoma-like) [24,25,26]. During this period, histotype reproducibility based on morphological criteria dramatically improved [21,27,28].

Changes regarding rare histotypes occurred in the fourth (2014) and fifth (2020) editions. The history of seromucinous tumors is particularly turbulent. Initially described as “mixed-epithelial papillary borderline tumors of Müllerian type”, this tumor was accepted by the third edition of the WHO Classification (2003) as “mucinous borderline tumor, endocervical type” [29,30]. The fourth edition separated it from intestinal-type mucinous tumors into its own category, recognizing its closer relationship to Müllerian-type epithelia (specifically, endometrioid tumors), and renamed it as “seromucinous”. Seromucinous tumors were allowed all three categories of benign seromucinous cystadenoma/adenofibroma, seromucinous borderline tumor and seromucinous carcinoma [31]. However, a subsequent study found that seromucinous carcinomas were not reproducibly diagnosable and immunohistochemically and molecularly could be reclassified into other histotypes, most often as ECs and some as LGSCs [32]. Therefore, the fifth edition of the WHO considers “seromucinous carcinoma” a variant of ECs (with mucinous differentiation), which should be distinguished from LGSCs (with mucinous differentiation). Seromucinous cystadenoma and borderline tumor remain as distinct categories.

With the evolution of ancillary diagnostic testing, the diagnosis of mixed carcinoma decreased dramatically [33]. It is now being recognized that phenotypical differences (morphological mimicry) within a tumor are a normal occurrence in tumors with intratumoral heterogeneity. Most morphologically mixed-appearing (including ambiguous) carcinomas can now be classified as one specific histotype. Nevertheless, rare exceptions to this rule exist, and, therefore, the fifth edition reintroduced mixed carcinoma, noting that these tumors are rare. The majority of mixed cases show a common clonal origin [33]. Mixed carcinomas are thought to develop via transdifferentiation of one Mullerian neoplasm to another or through divergence from a common precursor into two histotypes. Endometriosis-associated mixed EC/CCC are the most common scenario, favoring the latter possibility. Although shared mutations among the components of mixed carcinoma have been used to suggest such divergence, the recent finding of canonical cancer mutations in non-cancer-associated endometriosis [8,34] and even normal endometrium [35] suggests that the common mutations could reflect an origin from a mutant field (field effect) with histomorphologically normal cells, and tumors may have emerged from that field as independent and unrelated events. A more detailed and comprehensive review of the clonal relationships between mixed carcinoma elements and their surrounding normal tissue will be required to resolve this issue. Notably, rare carcinomas admixed with neuroendocrine carcinoma and pure primary ovarian neuroendocrine carcinomas do exist, though they are not listed in Table 1.

The new entity of mesonephric-like adenocarcinomas with similarities to mesonephric carcinoma of the uterine cervix was included in the fifth edition [36]. Based on associated Müllerian lesions and molecular findings, the current understanding is that these tumors arise from transdifferentiation of other Müllerian histotypes [37]. Mesonephric-like adenocarcinomas follow an aggressive clinical course, and almost all were historically diagnosed as endometrioid carcinomas [38,39]. Although ancillary IHC markers exist to support their diagnosis, the diagnostic distinction from endometrioid carcinoma remains challenging, and data for diagnostic reproducibility are not yet available. Molecularly, mesonephric-like adenocarcinomas are p53 normal, mismatch repair (MMR) proficient and frequently harbor *KRAS* mutations [37]. There is a case report on their endometrial counterpart showing sustained response to kinase inhibitors, indicating that the recognition of this unique histotype may be important for *KRAS*-targeted therapy [40].

Undifferentiated/dedifferentiated carcinomas are now molecularly characterized by SWItch/Sucrose Non-Fermentable (SWI/SNF) alterations. These tumors arise from endometrioid carcinomas, most commonly in a MMR-deficient (MMRd) background, by acquiring the following mutually exclusive alterations in the undifferentiated component: the co-mutation of *ARID1B/ARID1A*, *SMARCA4* (BRG1) or *SMARCB1* (INI1) [41]. These tumors are characterized by an extremely aggressive clinical course, often with progression under standard platinum–taxane chemotherapy [42]. Other tumors formerly diagnosed as undifferentiated carcinomas are currently more precisely classified as anaplastic carcinoma in mural nodules of a mucinous tumor, HGSC with solid morphology (SET features) or high-grade EC [21].

Carcinosarcomas are now considered of epithelial origin (metaplastic carcinoma), most frequently HGSC, and are therefore categorized as a malignant epithelial tumor rather than a mixed epithelial and mesenchymal tumor.

In summary, there are now five principal histotypes of malignant ovarian epithelial neoplasms, and, in descending order of frequency, they are HGSC, EC, CCC, LGSC and MC [43], as well as rare histotypes.

## 3. Ancillary Immunohistochemical Testing to Confirm a Morphological Histotype Diagnosis

**Figure 2:**
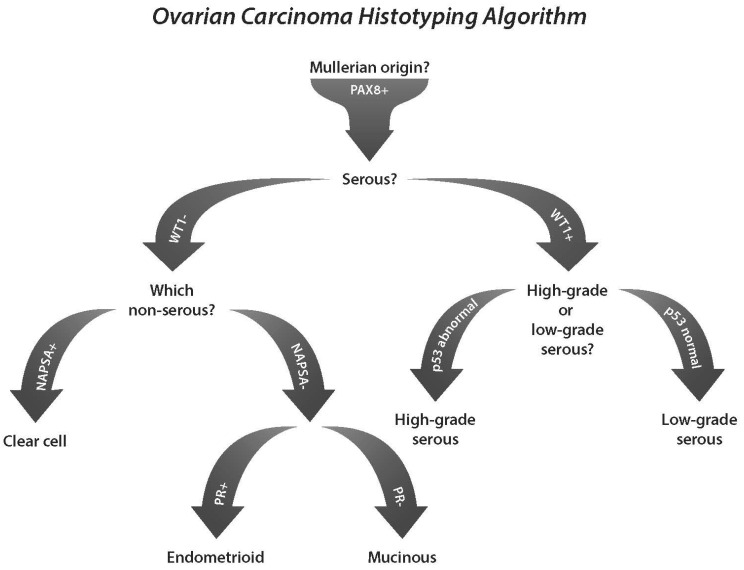
Four-marker immunohistochemical panel to distinguish the five principal histotypes of ovarian carcinomas: high-grade serous, low-grade serous, endometrioid, clear cell and mucinous carcinomas. PAX8 may be added as generic Mullerian marker, although there is limited sensitivity for endometrioid and mucinous carcinomas and limitations with specificity toward renal and thyroid primaries. NAPSA = napsin A.

After many iterations, we developed and validated a four-marker immunohistochemical panel that can distinguish the five principal histotypes with almost 90% precision (Figure 2) [3,23]. Given that morphological diagnosis also has ~90% accuracy [21,27], it can be expected that the integration of phenotypes with the current standard of ancillary IHC can achieve a diagnostic precision of >95%; however, this has not been formally tested. Notably, when ancillary diagnostic IHC was used on a post-2014 standardized pathology review, a subset of HGSC and MC was reclassified to EC (although not as many cases and not the same cases that were reclassified from the original pre-2014 diagnosis) [23]. This illustrates the potential for underdiagnosing EC when not using ancillary diagnostic IHC.

The specific use of ancillary IHC markers depends on the diagnostic considerations, which can be confirmatory (e.g., the characteristic combination of WT1 and p53 for HGSC and LGSC, Figure 1), exploratory (ambiguous morphology or research context: four-marker panel, Figure 2) or differential diagnostic (usually between two entities, Table 2).

The differential diagnostic approach between two entities is divided into first-line panels, which solve most of the cases and are sufficient if the morphological context is compatible, and more extensive second-line panels, which may be reserved for cases with phenotypes contradicting the first-line panel, unexpected first-line panel results or other unusual constellations. WT1 is the most important marker that is diffusely expressed in almost all HGSCs and LGSCs and virtually absent in almost all CCCs and MCs. However, it can be expressed in 10–15% of ECs [3,23]. Therefore, a combination of WT1 and p53 is the best panel to distinguish HGSC from EC [44]. Given the importance of an accurate diagnosis for targeted therapy with poly ADP ribose polymerase (PARP) inhibitors in high-grade serous carcinomas, predictive testing for both histotypes (*BRCA1/2* mutation status for HGSC and mismatch repair for EC) might be performed in rare high-grade cases that cannot be reliably classified. Serous carcinomas with moderate (grade 2) nuclear atypia may be subject to p53 IHC to distinguish HGSC from LGSC. This has only become possible after IHC optimization to accurately predict *TP53* mutation status [52,53]. The three-marker first-line panel of napsin A, HNF1B, and PR can aid in the distinction of CCC from EC, although this can be misleading in a few ECs with non-specific cytoplasmic clearing when the IHC panel suggests CCC. Accurate distinction requires the integration of morphology (underlying architecture: tubulocystic for CCC versus glandular for EC), IHC and genotype (MMRd for EC) [45]. The best markers to distinguish EC from MC are PR and vimentin [51]. MCs are notoriously difficult to distinguish from metastases from gastrointestinal primaries, but CK7 and SATB2 comprise a practical and accurate panel against metastasis from a lower gastrointestinal primary (colon/appendix) [54]. There is a need for ancillary tests to assist in the discrimination of ovarian MCs from metastatic adenocarcinomas originating from the upper gastrointestinal tract. Mesonephric-like adenocarcinomas are characterized by the expression of GATA3 or TTF1 with the absence of ER/PR expression. SWI/SNF-deficient dedifferentiated and undifferentiated carcinomas can be confirmed by the loss of ARID1B, BRG1 or INI1 by IHC.

## 4. Molecular Subtypes of Ovarian Carcinomas

### 4.1. High-Grade Serous Carcinoma

There are many ways to subclassify HGSCs [55]. HGSCs are morphologically heterogenous with many architectural patterns that can be simply categorized into papillary versus SET (solid, pseudoendometrioid and transitional cell carcinoma-like) [26]. While there is some phenotype–genotype correlation, it remains to be seen whether this is sufficiently precise to assist in further subclassification [56]. Bowtell and colleagues, and subsequently The Cancer Genome Atlas, described molecular subtypes of HGSC based on unsupervised clusters from mRNA expression data [57,58]. The Ovarian Tumor Tissue Analysis (OTTA) consortium consolidated these into four molecular subtypes (C1.MES, C2.IMM, C4.DIF and C5.PRO) using a 55 NanoString probe set [59]. This study is a nice example of scientific rigor and collaboration to establish a consensus molecular subtype based on mRNA expression while avoiding non-comparable results by individual approaches. The study also highlights the influence of anatomical sites on gene expression with signals coming from diverse tumor microenvironments. While molecular subtype conveys modest prognostic information, whether it can predict response to therapy remains to be determined.

Numerous studies have developed prognostic mRNA signatures for HGSC [60], but a recent large study from the OTTA consortium developed a 101-gene expression signature using the NanoString platform associated with a large effect size and median overall survival differences of more than seven years between quintiles [61]. This study shows the power of quantitative multigene signatures in better reflecting the complex cellular biology of HGSC. However, prognostic stratification has been validated for individual biomarkers, including the degree of CD8+ tumor-infiltrating lymphocytes, the level of PR expression and the presence of *CCNE1* high-level amplification (>eight copies) [62,63,64,65]. While prognostic information has no direct clinical value for a disease that is too aggressive to withhold adjuvant therapy even at the lowest stage, it provides insights into the biological behavior (prognosis) and response to therapy. Separating prognostic information from predictive information requires controlled clinical trials and can often only be inferred from observational cohort studies. For example, the recently described favorable association of the proliferation marker MCM3 with survival is thought to be due to good response to standard platinum–taxane chemotherapy [66]. Another example is the prognostic association of *BRCA1/2* mutations in patients, which could be at least partly due to a better response to platinum–taxane therapy [67]. Moreover, combinations of biomarkers seem to perform better than individual markers, as shown by the combination of homologous repair deficiency (HRD) and RB1 loss, which can predict long-term survival better than either alone [68].

HGSC is the prototype of a chromosomally unstable cancer. Brenton and colleagues defined seven distinct copy number signatures, each associated with a different mechanism of chromosomal instability [69]. Shah and colleagues proposed four major mechanisms of chromosomal instability, namely, *BRCA1*-associated tandem duplications, *BRCA2*-associated interstitial deletions, *CCNE1*-amplified associated fold-back inversions and *CDK12*-associated tandem duplications, and they showed that these are associated with different mechanisms of immune resistance, explaining the disappointing results in recent immune checkpoint inhibitor trials that recruited thousands of women diagnosed with HGSC [70,71].

The breakthrough for the treatment of HGSC was the recent approval of PARP inhibitors as a standard of care [72]. However, predicting the response for any given patient remains unresolved. This is reflected in the differences in companion diagnostics for different PARP inhibitors, ranging from clinical platinum sensitivity (agnostic of molecular tests) and *BRCA1/2* mutation status to commercial HRD tests [73]. Even the cut-offs for commercial HRD tests have been shifting, highlighting the challenges in establishing a threshold for a continuous variable that informs a binary treatment decision [73]. It remains to be seen whether signatures can reproducibly predict the response to PARP inhibitors [74,75], particularly since Brenton and colleagues have depicted the genomic entropy of HGSC with several copy number signatures present in any individual patient [69]. It may be worth considering giving PARP inhibitors to all patients with HGSC and then identifying the molecular characteristics of the patients that do not respond (negative predictive testing). This will likely identify patients with HR-proficient (HRP/non-HRD) tumors. HRP high-grade serous carcinomas are molecularly heterogeneous; a lead candidate for negative predictive testing is the presence of high-level amplifications of *CCNE1* given its mutual exclusivity to *BRCA1/2* germline mutations as shown by Bowtell and colleagues [64,65,76].

### 4.2. Endometrioid Carcinoma

One large study and other smaller studies have established that ovarian endometrioid carcinomas are composed of the same four molecular subtypes (*POLE* mutated, MMRd, no specific molecular profile (NSMP) and p53 abnormal (p53abn)) with the same prognostic stratification as their endometrial counterparts [77,78,79,80]. Patients whose tumors harbor a *POLE* mutation (*POLE*mut) have the most favorable prognosis, while patients with p53abn tumors can expect an aggressive disease course. MMRd and NSMP are associated with an intermediate prognosis. This stratification remained significant in uni- and multi-variable analyses when restricted to low-stage (defined as stages I–IIA) cases and provided better stratification than a histologic grade, providing further evidence that grading may eventually be replaced by molecular determinants. In contrast to the endometrium, however, the group of NSMP is substantially larger (73% versus 56%), requiring further stratification. The most promising biomarkers, which have only been assessed outside the context of molecular subtype thus far, are PR and *CTNNB1*, with the latter being the most commonly mutated gene in ovarian EC [45,46,63,81,82,83,84,85].

Treatment approaches for ovarian EC could be better aligned with their endometrial counterparts. Hormonal therapy may be considered for hormone receptor-positive endometrioid carcinomas that are not p53abn. MMR testing for Lynch syndrome screening should be performed, and patients with MMRd EC are eligible for immune checkpoint blockade therapy.

Of note, most historical cohorts still include poor prognostic un-/de-differentiated SWI/SNF-deficient carcinomas (mostly MMRd) and mesonephric-like adenocarcinomas (NSMP) in the group of endometrioid carcinomas. Excluding those and p53abn, a diagnosis of low-stage endometrioid carcinoma of other molecular subtypes without loss/reduced PR expression represents the best group for surveillance.

### 4.3. Clear Cell Carcinoma

Advanced clear cell carcinoma remains a therapeutic dilemma. Huntsman and colleagues discovered *ARID1A* mutations as the most common molecular alteration in CCC [86], but these are not independently prognostic [48]. ARID1A, as a regulatory subunit for the SWI/SNF complex, is a difficult therapeutic target [87]. Recent methylation analyses showed that clear cell carcinomas cluster into at least two broad groups (cluster 1 characterized by a high stage and *TP53* mutations and cluster 2 by co-occurring *ARID1A/PIK3CA* mutations and Asian ancestry) [88]. Gene expression and sequencing studies created two similar broad clusters [89]. Individual poor prognostic markers are p53, CDKN2A and IGF2BP3 [90,91]. Nevertheless, candidate biomarkers did not predict differing responses to standard platinum-based chemotherapy [92], and the search for therapeutic targets is ongoing. *ERBB2* amplifications occur in 7%–14% of CCCs, making it a good candidate for inclusion into basket trials [89,93].

Clinical trials and case reports suggest immune therapy; however, biomarker development to predict response to checkpoint inhibitor therapy has been challenging without consistent predictors (e.g., PD-L1 score) [94]. MMRd with a high tumor mutation burden and neoantigen expression are predictors of response to immune checkpoint blockade but do not explain all responsive cases. While there are obscure cases with diffuse intratumoral stromal inflammation that are MMRd and might be classified as CCC [95], MMRd does not occur in prototypical CCCs; hence, MMRd is better considered in the context of the endometrioid histotypes (see above) [45]. Recent studies evaluating the immune microenvironment of CCC suggest that tumor-associated macrophages may be a marker for immunosuppressive microenvironments [96,97]. Perhaps an overlay of the immune microenvironment with tumor intrinsic oncogenic alterations will better explain which patients respond to immune therapy.

### 4.4. Low-Grade Serous Carcinoma

Data from a large study integrating mutational data from targeted sequencing and IHC in LGSC suggest five molecular subtypes, and, listed in order of decreasing aggressiveness, they are CDKN2A IHC alteration > PR loss/high fraction of genome altered > MAPK pathway mutations (*KRAS*, *NRAS*, *BRAF*) ~ *USP9X* mutations ~ NSMP [98]. This could provide context for molecularly informed treatment decisions, such as CDK4/6 inhibitors for cases with CDKN2A loss versus hormonal therapy plus MEK inhibitors for MAPK-mutated cases with retained hormone receptor expression [63,90,99,100,101]. However, these findings require further validation in preclinical models and clinical trials.

### 4.5. Mucinous Carcinoma

The largest study of ovarian mucinous carcinomas confirmed frequent copy number losses (hetero- or homozygous) of *CDKN2A* and mutations in *CDKN2A* and *KRAS* as early events [102]. The progression from borderline tumor to carcinoma is often associated with the acquisition of a *TP53* mutation and additional copy number alterations [102,103]. In stark contrast to the dualistic pathway of serous carcinomas, *KRAS* and *TP53* mutations often co-occur in ovarian MC, perhaps explaining the resistance to platinum–taxane chemotherapy. Therapeutic options for advanced MC patients are practically non-existent, and current therapies are unlikely to be effective because HRD and MMRd do not occur [104]. The most promising target represents *ERRB2* amplification occurring in 26.7% in a recent study—all high level and focal, supported by IHC and often found in the context of a *TP53* mutation [104]. Despite a close phenotypical relationship to gastrointestinal tumors, it is now very clear that ovarian mucinous tumors are very different from lower gastrointestinal tumors and perhaps morphologically and molecularly closest to adenocarcinomas of the gastroesophageal junction. However, including ovarian MC into basket trials with specific biomarkers/biomarker combinations seems more promising than a simple cross-over of gastrointestinal treatment regimens.

## 5. Conclusions

The phenotypically informed histotype classification remains the mainstay of ovarian carcinoma subclassification. The histotype classification is particularly robust because histotypes arise from different cells of origin, allowing for cell lineage-specific diagnostic ancillary IHC markers in combination with histotype-specific oncogenic alterations. Ancillary IHC dramatically improves the precision of diagnostic histotyping.

The phenotype–genotype correlation has its limitations when it comes to molecular subtyping within histotypes. Phenotypes can direct certain tests [105], but most tests need to be carried out in a phenotype-agnostic manner specific for a given histotype (under the condition that other histotypes are vigorously excluded). There are many possible approaches to molecular subtyping. An integrated assessment of individual candidate biomarkers (mostly mutations and protein level) emerges for certain histotypes [106]. The chromosomal instability of HGSC represents a particular challenge, and it remains to be seen whether computational models of combinations of mutational information, mRNA expression data and protein levels can robustly predict treatment response. Since different mechanisms of chromosomal instability are associated with certain lead alterations, focusing on these (e.g., *CCNE1* and *CDK12*) is a pragmatic strategy for biomarker test development within clinical trials. While our understanding of the molecular composition of ovarian carcinomas has significantly advanced, the need for treatment options suitable for these alterations becomes more and more obvious. To expand the therapeutic spectrum, preclinical studies require histotype-defined and molecular subtype-characterized model systems [71].

## Figures and Tables

**Table 1:** Evolution of ovarian carcinoma histotypes in selected WHO Classifications of Female Genital Tumours.

WHO 1973, 1st ed.	WHO 2003, 3rd ed.	WHO 2014, 4th ed.	WHO 2020, 5th ed.
Serous	Serous	High-grade serous	High-grade serous
		Low-grade serous	Low-grade serous
Mucinous	Mucinous	Mucinous	Mucinous
		Seromucinous	
Endometrioid	Endometrioid	Endometrioid	Endometrioid
Clear cell	Clear cell	Clear cell	Clear cell
Brenner	Transitional cell	Brenner	Brenner
	Squamous		
			Mesonephric-like
Undifferentiated	Undifferentiated	Undifferentiated	Undifferentiated
			Carcinosarcoma
Mixed	Mixed		Mixed
Unclassified epithelial			

**Table 2:** First- and second-line immunohistochemical panels for differential diagnoses of two specific histotypes of ovarian carcinoma.

Histotype 1	Histotype 2	First-Line Panel	Second-Line Panel	Reference(s)
HGSC	EC	WT1/p53: WT1+/p53abn combination is 99% specific for HGSC. WT1-/p53 wild type is highly specific for EC. Note: 10–15% of ECs can be either WT1+ or p53abn (rarely, both).	MMR and ARID1A have limited sensitivity (12% and 25%, respectively) for EC but are specific. PR, ELAPOR1 have limited discriminatory values as they are present in 85% of ECs versus 40% of HGSCs. Nuclear CTNNB1 expression is specific for ECs and present in ~50%, mostly low-grade ECs with squamous differentiation. Consider testing for somatic *BRCA1/2* or HRD.	[3,44,45,46,47,48]
HGSC	LGSC	p53: p53abn excludes LGSC (100% specific); however, 2–4% of HGSCs can show p53 wild type staining despite harboring a *TP53* mutation due to a non-functional but expressed protein.	p16: in the context of p53 wild type staining, if p16 shows normal patchy/heterogeneous expression, the probability of LGSC is 84%; if p16 is block diffuse, the probability of HGSC is 88%. Rare cases of p53 wild type, p16 block diffuse LGSC do exist, but they seem to carry an adverse outcome. Consider sequencing for MAPK pathway mutations.	[49]
HGSC	CCC	WT1, napsin A, ER: WT1+/ER+ confirms HGSC. WT1-/napsin A+ confirms CCC.	HNF1B, ARID1A: some napsin A- CCCs are HNF1B+. ARID1A is lost in 42% of CCCs.	[3,48,50]
HGSC	MC	WT1: WT1+ confirms HGSC.		[3,23]
EC	LGSC	WT1: WT1+ alone has perfect sensitivity for LGSC but is expressed in 10–15% of ECs.	Specific markers for EC (CTNNB1, ARID1A, MMR).	[3]
EC	CCC	Napsin A, HNF1B, PR: napsin A+/HNF1B diffuse +/PR- supports CCC (note that areas of cytoplasmic clearing in EC can show this profile). Napsin A-/HNF1B non-diffuse/PR+ confirms EC.	ELAPOR1, CDX2, AMACR: ELAPOR1+, CDX2+, AMACR- support EC. Further, ambiguous or mixed EC/CCC or tumors with diffuse intratumoral stromal inflammation should be tested for MMR, and, if deficient, consider EC.	[45]
EC	MC	PR+ confirms EC, although 15% of ECs are PR-. Presence of any vimentin expression supports EC.	ER is usually negative in MC.	[51]
LGSC	CCC/MC	WT1: WT1+ in LGSC, WT1- in CCC/MC.		[3]
CCC	MC	Napsin A, mucin stain: napsin A+/mucin- in CCC. Napsin A-/mucin+ in MC.		[3]
EC	Meso- Nephric-like	GATA3, TTF1, ER, PR: GATA3+ and/or TTF1+ with ER-/PR- confirms mesonephric-like adenocarcinoma.		[38,39]
EC	DDC	ARID1B, BRG1, INI1: loss of any of these markers confirms DDC.		[41]

HGSC = high-grade serous carcinoma; EC = endometrioid carcinoma; CCC = clear cell carcinoma; LGSC = low-grade serous carcinoma; MC = mucinous carcinoma; DDC = dedifferentiated carcinoma; MMR = mismatch repair; p53abn = p53 abnormal; HRD = homologous repair deficiency. Generally, + means expression (i.e., any staining) is present; − means absent expression. Certain markers have specific cut-offs; please see References.

## References

[1] Köbel M., Kalloger S.E., Boyd N., McKinney S., Mehl E., Palmer C., Leung S., Bowen N.J., Ionescu D.N., Rajput A. (2008). Ovarian carcinoma subtypes are different diseases: Implications for biomarker studies. PLoS Med..

[2] Peres L.C., Cushing-Haugen K.L., Kobel M., Harris H.R., Berchuck A., Rossing M.A., Schildkraut J.M., Doherty J.A. (2019). Invasive Epithelial Ovarian Cancer Survival by Histotype and Disease Stage. J. Natl. Cancer Inst..

[3] Köbel M., Rahimi K., Rambau P.F., Naugler C., Le Page C., Meunier L., de Ladurantaye M., Lee S., Leung S., Goode E.L. (2016). An Immunohistochemical Algorithm for Ovarian Carcinoma Typing. Int. J. Gynecol. Pathol..

[4] Piek J.M., van Diest P.J., Zweemer R.P., Jansen J.W., Poort-Keesom R.J., Menko F.H., Gille J.J., Jongsma A.P., Pals G., Kenemans P. (2001). Dysplastic changes in prophylactically removed Fallopian tubes of women predisposed to developing ovarian cancer. J. Pathol..

[5] Lee Y., Miron A., Drapkin R., Nucci M.R., Medeiros F., Saleemuddin A., Garber J., Birch C., Mou H., Gordon R.W. (2007). A candidate precursor to serous carcinoma that originates in the distal fallopian tube. J. Pathol..

[6] Gilks C.B., Davidson B., Köbel M., Ledermann J.A., Lim D., Malpica A., Mikami Y., Singh N., Srinivasan R., Vang R. (2021). Ovary, Fallopian Tube and Primary Peritoneal Carcinoma Histopathology Reporting Guide.

[7] WHO (2020). Classification of Tumours Editorial Board. Female Genital Tumours.

[8] Anglesio M.S., Papadopoulos N., Ayhan A., Nazeran T.M., Noe M., Horlings H.M., Lum A., Jones S., Senz J., Seckin T. (2017). Cancer-Associated Mutations in Endometriosis without Cancer. N. Engl. J. Med..

[9] Cochrane D.R., Tessier-Cloutier B., Lawrence K.M., Nazeran T., Karnezis A.N., Salamanca C., Cheng A.S., McAlpine J.N., Hoang L.N., Gilks C.B. (2017). Clear cell and endometrioid carcinomas: Are their differences attributable to distinct cells of origin?. J. Pathol..

[10] Sah S., Fulmali R., McCluggage W.G. (2020). Low-grade Serous Carcinoma Arising in Inguinal Nodal Endosalpingiosis: Report of 2 Cases and Literature Review. Int. J. Gynecol. Pathol..

[11] McKenney J.K., Gilks C.B., Kalloger S., Longacre T.A. (2016). Classification of Extraovarian Implants in Patients With Ovarian Serous Borderline Tumors (Tumors of Low Malignant Potential) Based on Clinical Outcome. Am. J. Surg. Pathol..

[12] Scott S.A., Llaurado Fernandez M., Kim H., Elit L., Nourmoussavi M., Glaze S., Roberts L., Offman S.L., Rahimi K., Lytwyn A. (2020). Low-grade serous carcinoma (LGSC): A Canadian multicenter review of practice patterns and patient outcomes. Gynecol. Oncol..

[13] Wang Y., Wu R.C., Shwartz L.E., Haley L., Lin M.T., Shih Ie M., Kurman R.J. (2015). Clonality analysis of combined Brenner and mucinous tumours of the ovary reveals their monoclonal origin. J. Pathol..

[14] Kommoss F.K.F., Cheasley D., Wakefield M.J., Scott C.L., Campbell I.G., Gilks C.B., Gorringe K. (2021). Primary mucinous ovarian neoplasms rarely show germ cell histogenesis. Histopathology.

[15] De Leo A., Santini D., Ceccarelli C., Santandrea G., Palicelli A., Acquaviva G., Chiarucci F., Rosini F., Ravegnini G., Pession A. (2021). What Is New on Ovarian Carcinoma: Integrated Morphologic and Molecular Analysis Following the New 2020 World Health Organization Classification of Female Genital Tumors. Diagnostics.

[16] Serov S.F., Scully R.E., Sobin L.H. (1973). International Classification of Tumours. Histological Typing of Ovarian Tumours.

[17] Kurman R.J., Carcangiu M.L., Herrington C.S., Young R.H. (2014). WHO Classification of Tumours of Female Reproductive Organs.

[18] Singer G., Oldt R., Cohen Y., Wang B.G., Sidransky D., Kurman R.J., Shih Ie M. (2003). Mutations in BRAF and KRAS characterize the development of low-grade ovarian serous carcinoma. J. Natl. Cancer Inst..

[19] Ahmed A.A., Etemadmoghadam D., Temple J., Lynch A.G., Riad M., Sharma R., Stewart C., Fereday S., Caldas C., Defazio A. (2010). Driver mutations in TP53 are ubiquitous in high grade serous carcinoma of the ovary. J. Pathol..

[20] Brett M.A., Llaurado Fernandez M., Langlais E., Tone A., Ghatage P., Glaze S., Provencher D., Rahimi K., Offman S.L., Scott S.A. (2020). Low-grade serous ovarian carcinoma: Recommendation for efficient ancillary testing and standardized biomarker reporting from the Canadian LGSC community of practice. Can. J. Pathol..

[21] Kommoss S., Gilks C.B., du Bois A., Kommoss F. (2016). Ovarian carcinoma diagnosis: The clinical impact of 15 years of change. Br. J. Cancer.

[22] Peres L.C., Cushing-Haugen K.L., Anglesio M., Wicklund K., Bentley R., Berchuck A., Kelemen L.E., Nazeran T.M., Gilks C.B., Harris H.R. (2018). Histotype classification of ovarian carcinoma: A comparison of approaches. Gynecol. Oncol..

[23] Köbel M., Luo L., Grevers X., Lee S., Brooks-Wilson A., Gilks C.B., Le N.D., Cook L.S. (2019). Ovarian Carcinoma Histotype: Strengths and Limitations of Integrating Morphology With Immunohistochemical Predictions. Int. J. Gynecol. Pathol..

[24] Schwartz D.R., Kardia S.L., Shedden K.A., Kuick R., Michailidis G., Taylor J.M., Misek D.E., Wu R., Zhai Y., Darrah D.M. (2002). Gene expression in ovarian cancer reflects both morphology and biological behavior, distinguishing clear cell from other poor-prognosis ovarian carcinomas. Cancer Res..

[25] Acs G., Pasha T., Zhang P.J. (2004). WT1 is differentially expressed in serous, endometrioid, clear cell, and mucinous carcinomas of the peritoneum, fallopian tube, ovary, and endometrium. Int. J. Gynecol. Pathol..

[26] Soslow R.A., Han G., Park K.J., Garg K., Olvera N., Spriggs D.R., Kauff N.D., Levine D.A. (2012). Morphologic patterns associated with BRCA1 and BRCA2 genotype in ovarian carcinoma. Mod. Pathol..

[27] Köbel M., Kalloger S.E., Baker P.M., Ewanowich C.A., Arseneau J., Zherebitskiy V., Abdulkarim S., Leung S., Duggan M.A., Fontaine D. (2010). Diagnosis of ovarian carcinoma cell type is highly reproducible: A transcanadian study. Am. J. Surg. Pathol..

[28] Köbel M., Bak J., Bertelsen B.I., Carpen O., Grove A., Hansen E.S., Levin Jakobsen A.M., Lidang M., Masback A., Tolf A. (2014). Ovarian carcinoma histotype determination is highly reproducible, and is improved through the use of immunohistochemistry. Histopathology.

[29] Rutgers J.L., Scully R.E. (1988). Ovarian mixed-epithelial papillary cystadenomas of borderline malignancy of mullerian type. A clinicopathologic analysis. Cancer.

[30] Tavassoli F.A., Devilee P. (2003). WHO Classification of Tumours. Tumors of the Breast and Female Genital Organs.

[31] Taylor J., McCluggage W.G. (2015). Ovarian seromucinous carcinoma: Report of a series of a newly categorized and uncommon neoplasm. Am. J. Surg. Pathol..

[32] Rambau P.F., McIntyre J.B., Taylor J., Lee S., Ogilvie T., Sienko A., Morris D., Duggan M.A., McCluggage W.G., Kobel M. (2017). Morphologic Reproducibility, Genotyping, and Immunohistochemical Profiling Do Not Support a Category of Seromucinous Carcinoma of the Ovary. Am. J. Surg. Pathol..

[33] Mackenzie R., Talhouk A., Eshragh S., Lau S., Cheung D., Chow C., Le N., Cook L.S., Wilkinson N., McDermott J. (2015). Morphologic and Molecular Characteristics of Mixed Epithelial Ovarian Cancers. Am. J. Surg. Pathol..

[34] Lac V., Verhoef L., Aguirre-Hernandez R., Nazeran T.M., Tessier-Cloutier B., Praetorius T., Orr N.L., Noga H., Lum A., Khattra J. (2019). Iatrogenic endometriosis harbors somatic cancer-driver mutations. Hum. Reprod..

[35] Lac V., Nazeran T.M., Tessier-Cloutier B., Aguirre-Hernandez R., Albert A., Lum A., Khattra J., Praetorius T., Mason M., Chiu D. (2019). Oncogenic mutations in histologically normal endometrium: The new normal?. J. Pathol..

[36] McFarland M., Quick C.M., McCluggage W.G. (2016). Hormone receptor-negative, thyroid transcription factor 1-positive uterine and ovarian adenocarcinomas: Report of a series of mesonephric-like adenocarcinomas. Histopathology.

[37] da Silva E.M., Fix D.J., Sebastiao A.P.M., Selenica P., Ferrando L., Kim S.H., Stylianou A., Da Cruz Paula A., Pareja F., Smith E.S. (2021). Mesonephric and mesonephric-like carcinomas of the female genital tract: Molecular characterization including cases with mixed histology and matched metastases. Mod. Pathol..

[38] Pors J., Segura S., Chiu D.S., Almadani N., Ren H., Fix D.J., Howitt B.E., Kolin D., McCluggage W.G., Mirkovic J. (2021). Clinicopathologic Characteristics of Mesonephric Adenocarcinomas and Mesonephric-like Adenocarcinomas in the Gynecologic Tract: A Multi-institutional Study. Am. J. Surg. Pathol..

[39] Kang E.Y., Rodriguez M., Lee S., Wiebe N., Liu Y., Cook L.S., Lee C.H., Karnezis A., Köbel M. (2021). Abstracts from USCAP 2021: Gynecologic And Obstetric Pathology: Mesonephric-Like Carcinoma of the Ovary is a Rare and Aggressive Histotype of Ovarian Carcinoma (582). Lab. Investig..

[40] Shen S., Rubinstein M.M., Park K.J., Konner J.A., Makker V. (2021). Sustained response to lenvatinib and pembrolizumab in two patients with KRAS-mutated endometrial mesonephric-like adenocarcinoma. Gynecol. Oncol. Rep..

[41] Coatham M., Li X., Karnezis A.N., Hoang L.N., Tessier-Cloutier B., Meng B., Soslow R.A., Blake Gilks C., Huntsman D.G., Stewart C.J. (2016). Concurrent ARID1A and ARID1B inactivation in endometrial and ovarian dedifferentiated carcinomas. Mod. Pathol..

[42] Tessier-Cloutier B., Coatham M., Carey M., Nelson G.S., Hamilton S., Lum A., Soslow R.A., Stewart C.J., Postovit L.M., Köbel M. (2021). SWI/SNF-deficiency defines highly aggressive undifferentiated endometrial carcinoma. J. Pathol. Clin. Res..

[43] Köbel M., Kalloger S.E., Huntsman D.G., Santos J.L., Swenerton K.D., Seidman J.D., Gilks C.B. (2010). Differences in tumor type in low-stage versus high-stage ovarian carcinomas. Int. J. Gynecol. Pathol..

[44] Assem H., Rambau P.F., Lee S., Ogilvie T., Sienko A., Kelemen L.E., Köbel M. (2018). High-grade Endometrioid Carcinoma of the Ovary: A Clinicopathologic Study of 30 Cases. Am. J. Surg. Pathol..

[45] Rodriguez M., Kang E.Y., Farrington K., Cook L.S., Le N.D., Karnezis A.N., Lee C.H., Nelson G.S., Terzic T., Lee S. (2021). Accurate Distinction of Ovarian Clear Cell From Endometrioid Carcinoma Requires Integration of Phenotype, Immunohistochemical Predictions, and Genotype: Implications for Lynch Syndrome Screening. Am. J. Surg. Pathol..

[46] Wang L., Rambau P.F., Kelemen L.E., Anglesio M.S., Leung S., Talhouk A., Köbel M. (2019). Nuclear β-catenin and CDX2 expression in ovarian endometrioid carcinoma identify patients with favourable outcome. Histopathology.

[47] Dieters-Castator D.Z., Rambau P.F., Kelemen L.E., Siegers G.M., Lajoie G.A., Postovit L.M., Köbel M. (2019). Proteomics-Derived Biomarker Panel Improves Diagnostic Precision to Classify Endometrioid and High-grade Serous Ovarian Carcinoma. Clin. Cancer Res..

[48] Heinze K., Nazeran T.M., Lee S., Krämer P., Cairns E.S., Chiu D.S., Leung S.C.Y., Kang E.Y., Meagher N.S., Kennedy C.J. (2021). Validated biomarker assays confirm ARID1A loss is confounded with MMR deficiency, CD8 TIL infiltration, and provides no independent prognostic value in endometriosis-associated ovarian carcinomas. J. Pathol..

[49] Altman A.D., Nelson G.S., Ghatage P., McIntyre J.B., Capper D., Chu P., Nation J.G., Karnezis A.N., Han G., Kalloger S.E. (2013). The diagnostic utility of TP53 and CDKN2A to distinguish ovarian high-grade serous carcinoma from low-grade serous ovarian tumors. Mod. Pathol..

[50] Köbel M., Kalloger S.E., Carrick J., Huntsman D., Asad H., Oliva E., Ewanowich C.A., Soslow R.A., Gilks C.B. (2009). A limited panel of immunomarkers can reliably distinguish between clear cell and high-grade serous carcinoma of the ovary. Am. J. Surg. Pathol..

[51] Woodbeck R., Kelemen L.E., Köbel M. (2019). Ovarian Endometrioid Carcinoma Misdiagnosed as Mucinous Carcinoma: An Underrecognized Problem. Int. J. Gynecol. Pathol..

[52] Köbel M., Piskorz A.M., Lee S., Lui S., LePage C., Marass F., Rosenfeld N., Mes Masson A.M., Brenton J.D. (2016). Optimized p53 immunohistochemistry is an accurate predictor of TP53 mutation in ovarian carcinoma. J. Pathol. Clin. Res..

[53] Köbel M., Kang E.Y. (2021). The Many Uses of p53 Immunohistochemistry in Gynecological Pathology: Proceedings of the ISGyP Companion Society Session at the 2020 USCAP Annual9 Meeting. Int. J. Gynecol. Pathol..

[54] Meagher N.S., Wang L., Rambau P.F., Intermaggio M.P., Huntsman D.G., Wilkens L.R., El-Bahrawy M.A., Ness R.B., Odunsi K., Steed H. (2019). A combination of the immunohistochemical markers CK7 and SATB2 is highly sensitive and specific for distinguishing primary ovarian mucinous tumors from colorectal and appendiceal metastases. Mod. Pathol..

[55] Hatano Y., Hatano K., Tamada M., Morishige K.I., Tomita H., Yanai H., Hara A. (2019). A Comprehensive Review of Ovarian Serous Carcinoma. Adv. Anat. Pathol..

[56] Murakami R., Matsumura N., Mandai M., Yoshihara K., Tanabe H., Nakai H., Yamanoi K., Abiko K., Yoshioka Y., Hamanishi J. (2016). Establishment of a Novel Histopathological Classification of High-Grade Serous Ovarian Carcinoma Correlated with Prognostically Distinct Gene Expression Subtypes. Am. J. Pathol..

[57] Tothill R.W., Tinker A.V., George J., Brown R., Fox S.B., Lade S., Johnson D.S., Trivett M.K., Etemadmoghadam D., Locandro B. (2008). Novel molecular subtypes of serous and endometrioid ovarian cancer linked to clinical outcome. Clin. Cancer Res..

[58] Cancer Genome Atlas Research N. (2011). Integrated genomic analyses of ovarian carcinoma. Nature.

[59] Talhouk A., George J., Wang C., Budden T., Tan T.Z., Chiu D.S., Kommoss S., Leong H.S., Chen S., Intermaggio M.P. (2020). Development and Validation of the Gene Expression Predictor of High-grade Serous Ovarian Carcinoma Molecular SubTYPE (PrOTYPE). Clin. Cancer Res..

[60] Verhaak R.G., Tamayo P., Yang J.Y., Hubbard D., Zhang H., Creighton C.J., Fereday S., Lawrence M., Carter S.L., Mermel C.H. (2013). Prognostically relevant gene signatures of high-grade serous ovarian carcinoma. J. Clin. Investig..

[61] Millstein J., Budden T., Goode E.L., Anglesio M.S., Talhouk A., Intermaggio M.P., Leong H.S., Chen S., Elatre W., Gilks B. (2020). Prognostic gene expression signature for high-grade serous ovarian cancer. Ann. Oncol..

[62] Goode E.L., Block M.S., Kalli K.R., Vierkant R.A., Chen W., Fogarty Z.C., Gentry-Maharaj A., Toloczko A., Hein A., Ovarian Tumor Tissue Analysis Consortium (2017). Dose-Response Association of CD8+ Tumor-Infiltrating Lymphocytes and Survival Time in High-Grade Serous Ovarian Cancer. JAMA Oncol..

[63] Sieh W., Köbel M., Longacre T.A., Bowtell D.D., deFazio A., Goodman M.T., Høgdall E., Deen S., Wentzensen N., Moysich K.B. (2013). Hormone-receptor expression and ovarian cancer survival: An Ovarian Tumor Tissue Analysis consortium study. Lancet Oncol..

[64] Etemadmoghadam D., deFazio A., Beroukhim R., Mermel C., George J., Getz G., Tothill R., Okamoto A., Raeder M.B., Harnett P. (2009). Integrated genome-wide DNA copy number and expression analysis identifies distinct mechanisms of primary chemoresistance in ovarian carcinomas. Clin. Cancer Res..

[65] Chan A.M., Enwere E., McIntyre J.B., Wilson H., Nwaroh C., Wiebe N., Ou Y., Liu S., Wiedemeyer K., Rambau P.F. (2020). Combined CCNE1 high-level amplification and overexpression is associated with unfavourable outcome in tubo-ovarian high-grade serous carcinoma. J. Pathol. Clin. Res..

[66] Kang E.Y., Millstein J., Popovic G., Meagher N.S., Bolithon A., Talhouk A., Chiu D.S., Anglesio M.S., Leung B., Tang K. (2021). MCM3 is a novel proliferation marker associated with longer survival for patients with tubo-ovarian high-grade serous carcinoma. Virchows Arch..

[67] Bolton K.L., Chenevix-Trench G., Goh C., Sadetzki S., Ramus S.J., Karlan B.Y., Lambrechts D., Despierre E., Barrowdale D., McGuffog L. (2012). Association between BRCA1 and BRCA2 mutations and survival in women with invasive epithelial ovarian cancer. JAMA.

[68] Garsed D.W., Alsop K., Fereday S., Emmanuel C., Kennedy C.J., Etemadmoghadam D., Gao B., Gebski V., Gares V., Christie E.L. (2018). Homologous Recombination DNA Repair Pathway Disruption and Retinoblastoma Protein Loss Are Associated with Exceptional Survival in High-Grade Serous Ovarian Cancer. Clin. Cancer Res..

[69] Macintyre G., Goranova T.E., De Silva D., Ennis D., Piskorz A.M., Eldridge M., Sie D., Lewsley L.A., Hanif A., Wilson C. (2018). Copy number signatures and mutational processes in ovarian carcinoma. Nat. Genet..

[70] Vázquez-García I., Uhlitz F., Ceglia N., Lim J.L.P., Wu M., Mohibullah N., Ruiz A.E.B., Boehm K.M., Bojilova V., Fong J.F. (2021). Immune and malignant cell phenotypes of ovarian cancer are determined by distinct mutational processes. bioRxiv.

[71] Virani S., Baiocchi G., Bowtell D., Cabasag C.J., Cho K.R., Fortner R.T., Fujiwara K., Kim J.W., Köbel M., Kurtz J.E. (2021). Joint IARC/NCI International Cancer Seminar Series Report: Expert consensus on future directions for ovarian carcinoma research. Carcinogenesis.

[72] Lord C.J., Ashworth A. (2017). PARP inhibitors: Synthetic lethality in the clinic. Science.

[73] Funingana I.G., Reinius M.A.V., Petrillo A., Ang J.E., Brenton J.D. (2021). Can integrative biomarker approaches improve prediction of platinum and PARP inhibitor response in ovarian cancer?. Semin. Cancer Biol..

[74] van Wijk L.M., Vermeulen S., Meijers M., van Diest M.F., Ter Haar N.T., de Jonge M.M., Solleveld-Westerink N., van Wezel T., van Gent D.C., Kroep J.R. (2020). The RECAP Test Rapidly and Reliably Identifies Homologous Recombination-Deficient Ovarian Carcinomas. Cancers.

[75] van Wijk L.M., Kramer C.J.H., Vermeulen S., Ter Haar N.T., de Jonge M.M., Kroep J.R., de Kroon C.D., Gaarenstroom K.N., Vrieling H., Bosse T. (2021). The RAD51-FFPE Test; Calibration of a Functional Homologous Recombination Deficiency Test on Diagnostic Endometrial and Ovarian Tumor Blocks. Cancers.

[76] Etemadmoghadam D., Weir B.A., Au-Yeung G., Alsop K., Mitchell G., George J., Davis S., D'Andrea A.D., Simpson K., Hahn W.C. (2013). Synthetic lethality between CCNE1 amplification and loss of BRCA1. Proc. Natl. Acad. Sci. USA.

[77] Kramer P., Talhouk A., Brett M.A., Chiu D.S., Cairns E.S., Scheunhage D.A., Hammond R.F.L., Farnell D., Nazeran T.M., Grube M. (2020). Endometrial Cancer Molecular Risk Stratification is Equally Prognostic for Endometrioid Ovarian Carcinoma. Clin. Cancer Res..

[78] Cancer Genome Atlas Research N., Kandoth C., Schultz N., Cherniack A.D., Akbani R., Liu Y., Shen H., Robertson A.G., Pashtan I., Shen R. (2013). Integrated genomic characterization of endometrial carcinoma. Nature.

[79] Parra-Herran C., Lerner-Ellis J., Xu B., Khalouei S., Bassiouny D., Cesari M., Ismiil N., Nofech-Mozes S. (2017). Molecular-based classification algorithm for endometrial carcinoma categorizes ovarian endometrioid carcinoma into prognostically significant groups. Mod. Pathol..

[80] Leskela S., Romero I., Rosa-Rosa J.M., Caniego-Casas T., Cristobal E., Pérez-Mies B., Gutierrez-Pecharroman A., Santón A., Ojeda B., López-Reig R. (2020). Molecular Heterogeneity of Endometrioid Ovarian Carcinoma: An Analysis of 166 Cases Using the Endometrial Cancer Subrogate Molecular Classification. Am. J. Surg. Pathol..

[81] Rambau P., Kelemen L.E., Steed H., Quan M.L., Ghatage P., Kobel M. (2017). Association of Hormone Receptor Expression with Survival in Ovarian Endometrioid Carcinoma: Biological Validation and Clinical Implications. Int. J. Mol. Sci..

[82] Rambau P.F., Duggan M.A., Ghatage P., Warfa K., Steed H., Perrier R., Kelemen L.E., Kobel M. (2016). Significant frequency of MSH2/MSH6 abnormality in ovarian endometrioid carcinoma supports histotype-specific Lynch syndrome screening in ovarian carcinomas. Histopathology.

[83] Hollis R.L., Stanley B., Iida Y., Thomson J., Churchman M., Rye T., Mackean M., Nussey F., Gourley C., Herrington C.S. (2019). Hormone receptor expression patterns define clinically meaningful subgroups of endometrioid ovarian carcinoma. Gynecol. Oncol..

[84] Hollis R.L., Thomson J.P., Stanley B., Churchman M., Meynert A.M., Rye T., Bartos C., Iida Y., Croy I., Mackean M. (2020). Molecular stratification of endometrioid ovarian carcinoma predicts clinical outcome. Nat. Commun..

[85] Hollis R.L., Stanley B., Thomson J.P., Churchman M., Croy I., Rye T., Bartos C., Nussey F., Mackean M., Meynert A.M. (2021). Integrated molecular characterisation of endometrioid ovarian carcinoma identifies opportunities for stratification. NPJ Precis. Oncol..

[86] Wiegand K.C., Shah S.P., Al-Agha O.M., Zhao Y., Tse K., Zeng T., Senz J., McConechy M.K., Anglesio M.S., Kalloger S.E. (2010). ARID1A mutations in endometriosis-associated ovarian carcinomas. N. Engl. J. Med..

[87] Takahashi K., Takenaka M., Okamoto A., Bowtell D.D.L., Kohno T. (2021). Treatment Strategies for ARID1A-Deficient Ovarian Clear Cell Carcinoma. Cancers.

[88] Cunningham J.M., Winham S.J., Wang C., Weigelt B., Fu Z., Armasu S.M., McCauley B.M., Brand A.H., Chiew Y.E., Elishaev E. (2021). DNA Methylation Profiles of Ovarian Clear Cell Carcinoma. Cancer Epidemiol. Biomark. Prev..

[89] Tan D.S., Iravani M., McCluggage W.G., Lambros M.B., Milanezi F., Mackay A., Gourley C., Geyer F.C., Vatcheva R., Millar J. (2011). Genomic analysis reveals the molecular heterogeneity of ovarian clear cell carcinomas. Clin. Cancer Res..

[90] Rambau P.F., Vierkant R.A., Intermaggio M.P., Kelemen L.E., Goodman M.T., Herpel E., Pharoah P.D., Kommoss S., Jimenez-Linan M., Karlan B.Y. (2018). Association of p16 expression with prognosis varies across ovarian carcinoma histotypes: An Ovarian Tumor Tissue Analysis consortium study. J. Pathol. Clin. Res..

[91] Liu H., Zeng Z., Afsharpad M., Lin C., Wang S., Yang H., Liu S., Kelemen L.E., Xu W., Ma W. (2019). Overexpression of IGF2BP3 as a Potential Oncogene in Ovarian Clear Cell Carcinoma. Front. Oncol..

[92] Takenaka M., Kobel M., Garsed D.W., Fereday S., Pandey A., Etemadmoghadam D., Hendley J., Kawabata A., Noguchi D., Yanaihara N. (2019). Survival Following Chemotherapy in Ovarian Clear Cell Carcinoma Is Not Associated with Pathological Misclassification of Tumor Histotype. Clin. Cancer Res..

[93] Wiedemeyer K., Wang L., Kang E.Y., Liu S., Ou Y., Kelemen L.E., Feil L., Anglesio M.S., Glaze S., Ghatage P. (2021). Prognostic and Theranostic Biomarkers in Ovarian Clear Cell Carcinoma. Int. J. Gynecol. Pathol..

[94] Lin Y.C., Wen K.C., Sung P.L., Chou Y.T., Liew P.L., Chen L.Y., Huang R.L., Lai H.C., Chang L.T. (2020). Complete remission of heavily treated ovarian clear cell carcinoma with ARID1A mutations after pembrolizumab and bevacizumab combination therapy: A case report. J. Ovarian Res..

[95] Bennett J.A., Morales-Oyarvide V., Campbell S., Longacre T.A., Oliva E. (2016). Mismatch Repair Protein Expression in Clear Cell Carcinoma of the Ovary: Incidence and Morphologic Associations in 109 Cases. Am. J. Surg. Pathol..

[96] Sue A.Q.R., Patel P.G., Shakfa N., Nyi M.N., Afriyie-Asante A., Kang E.Y., Köbel M., Koti M. (2021). Prognostic significance of T cells, PD-L1 immune checkpoint and tumour associated macrophages in clear cell carcinoma of the ovary. Gynecol. Oncol..

[97] Khalique S., Nash S., Mansfield D., Wampfler J., Attygale A., Vroobel K., Kemp H., Buus R., Cottom H., Roxanis I. (2021). Quantitative Assessment and Prognostic Associations of the Immune Landscape in Ovarian Clear Cell Carcinoma. Cancers.

[98] Cheasley D., Nigam A., Zethoven M., Hunter S., Etemadmoghadam D., Semple T., Allan P., Carey M.S., Fernandez M.L., Dawson A. (2021). Genomic analysis of low-grade serous ovarian carcinoma to identify key drivers and therapeutic vulnerabilities. J. Pathol..

[99] Llaurado Fernandez M., Dawson A., Kim H., Lam N., Russell H., Bruce M., Bittner M., Hoenisch J., Scott S.A., Talhouk A. (2020). Hormone receptor expression and outcomes in low-grade serous ovarian carcinoma. Gynecol. Oncol..

[100] Shrestha R., Llaurado Fernandez M., Dawson A., Hoenisch J., Volik S., Lin Y.Y., Anderson S., Kim H., Haegert A.M., Colborne S. (2021). Multiomics Characterization of Low-Grade Serous Ovarian Carcinoma Identifies Potential Biomarkers of MEK Inhibitor Sensitivity and Therapeutic Vulnerability. Cancer Res..

[101] Chui M.H., Chang J.C., Zhang Y., Zehir A., Schram A.M., Konner J., Drilon A.E., Da Cruz Paula A., Weigelt B., Grisham R.N. (2021). Spectrum of BRAF Mutations and Gene Rearrangements in Ovarian Serous Carcinoma. JCO Precis. Oncol..

[102] Cheasley D., Wakefield M.J., Ryland G.L., Allan P.E., Alsop K., Amarasinghe K.C., Ananda S., Anglesio M.S., Au-Yeung G., Bohm M. (2019). The molecular origin and taxonomy of mucinous ovarian carcinoma. Nat. Commun..

[103] Kang E.Y., Cheasley D., LePage C., Wakefield M.J., da Cunha Torres M., Rowley S., Salazar C., Xing Z., Allan P., Bowtell D.D.L. (2021). Refined cut-off for TP53 immunohistochemistry improves prediction of TP53 mutation status in ovarian mucinous tumors: Implications for outcome analyses. Mod. Pathol..

[104] Gorringe K.L., Cheasley D., Wakefield M.J., Ryland G.L., Allan P.E., Alsop K., Amarasinghe K.C., Ananda S., Bowtell D.D.L., Christie M. (2020). Therapeutic options for mucinous ovarian carcinoma. Gynecol. Oncol..

[105] Kang E.Y., Wiebe N.J., Aubrey C., Lee C.H., Anglesio M.S., Tilley D., Ghatage P., Nelson G.S., Lee S., Köbel M. (2022). Selection of endometrial carcinomas for p53 immunohistochemistry based on nuclear features. J. Pathol. Clin. Res..

[106] Santandrea G., Piana S., Valli R., Zanelli M., Gasparini E., De Leo A., Mandato V.D., Palicelli A. (2021). Immunohistochemical Biomarkers as a Surrogate of Molecular Analysis in Ovarian Carcinomas: A Review of the Literature. Diagnostics.

